# Dogs and humans respond to emotionally competent stimuli by producing different facial actions

**DOI:** 10.1038/s41598-017-15091-4

**Published:** 2017-11-14

**Authors:** Cátia Caeiro, Kun Guo, Daniel Mills

**Affiliations:** 1 School of Psychology, University of Lincoln, Lincoln, UK; 2 School of Life Sciences, University of Lincoln, Lincoln, UK

## Abstract

The commonality of facial expressions of emotion has been studied in different species since Darwin, with most of the research focusing on closely related primate species. However, it is unclear to what extent there exists common facial expression in species more phylogenetically distant, but sharing a need for common interspecific emotional understanding. Here we used the objective, anatomically-based tools, FACS and DogFACS (Facial Action Coding Systems), to quantify and compare human and domestic dog facial expressions in response to emotionally-competent stimuli associated with different categories of emotional arousal. We sought to answer two questions: Firstly, do dogs display specific discriminatory facial movements in response to different categories of emotional stimuli? Secondly, do dogs display similar facial movements to humans when reacting in emotionally comparable contexts? We found that dogs displayed distinctive facial actions depending on the category of stimuli. However, dogs produced different facial movements to humans in comparable states of emotional arousal. These results refute the commonality of emotional expression across mammals, since dogs do not display human-like facial expressions. Given the unique interspecific relationship between dogs and humans, two highly social but evolutionarily distant species sharing a common environment, these findings give new insight into the origin of emotion expression.

## Introduction

The common origin of emotions has long been a subject of scientific interest^1^ with different emotional responses producing a diverse range of communicative elements, especially through the face. Facial expressions are also correlates of internal state in both humans^2^ and other animals^3–6^ and so may be used, in part, to infer emotion alongside other component processes, such as physiological activation and behavioural tendencies^7^. Many studies (e.g.^8,9^) use an holistic approach (i.e. categorizing the whole face as angry, happy, etc.) to classify the target facial expressions, which reflects the way the human brain processes faces^7,10^, but can be problematic when examining the underlying mechanism of emotion perception across species. For instance, there is a diverse range of smiling faces with different visual characteristics and different emotional meanings in humans^11,12^. As a classic example, the Duchenne smile (felt, true enjoyment) differs by one muscle contraction from the non-Duchenne smile (unfelt, usually produced in formal greetings). Moreover, during laughter and depending on the context, a blend of both Duchenne and non-Duchenne smiles is often observed^13^. Hence, simply classifying a facial expression as “happy” is too simplistic and less meaningful for cross-species comparison. Furthermore, the same ‘holistic’ facial morphological configuration could have different functional meanings (i.e. result in distinctly different behavioural consequences) depending on the species^14,15^. For example, the Play Face (PF) and the Full Play Face (FPF) are variants of the same facial expression, where the former presents an open mouth with lower teeth exposed, and the latter incorporates visible upper teeth. Both the PF and the FPF represent different degrees of playful expression in great apes (humans included)^16–18^. Conversely, in crested macaques, mandrills and geladas, the FPF is not just a more intense version of the PF, but instead is derived from convergence between the PF and the silent-bared teeth display (SBT), a facial expression observed in affiliative settings such as grooming^19^. Additionally, the SBT indicates submission and appeasement in Barbary macaques^14^, signals affinity and benign intentions in humans, and, in chimpanzees, is present in a range of situations from response to aggression to affinity contexts^15^.

As an alternative to an holistic descriptive approach, the decomposition and objective description of distinct anatomical regions of facial features, such as occurs with the Facial Action Coding System (FACS^20^), has been the golden standard to study human facial expressions of emotion across individuals of different races and cultures for several decades^21,22^. Each of the discrete facial movements identified (Action Units, AUs) is the result of an independent facial muscle contraction that can produce several changes in appearance to the face, which in turn are used to identify which AUs are activated. Thus, FACS codes facial movements from a purely anatomical basis, avoiding circular reasoning or *a priori* assumptions of emotion meaning. Recently, FACS has been adapted to several non-human species^23–30^, such as chimpanzees and orangutans, following the original methodology^20^ and has proven to be a successful tool for objectively investigating and comparing facial expressions of closely related species^31,32^. For example, chimpanzees and humans share an identical facial muscle plan (differing by only one muscle)^20,33^, but chimpanzees display both homologous (e.g. play face and human laugh) and species-specific expressions (e.g. pant-hoot)^32,34^.

While the human prototypical facial expressions of emotion are well established, little is known about the quantitative and empirical nature of the emotional facial displays of the domestic dog, an evolutionarily remote, but socially complex species which often shares the human social environment and frequently engages in interspecific communication with an emotional content (e.g.^35^). To date, functional facial expressions in dogs have been largely discussed holistically in relation to their approach-avoidance value, for example, the “threat gape” in fight-flight situations^3^, and the PF or the Relaxed Open Mouth (ROM) as a social communicative signal for play solicitation and within play bouts^3,36,37^. With the development of the FACS for the domestic dog^24^, it becomes possible to apply a bottom-up technique to investigate the composition and meaning of dogs’ facial expressions and, more importantly, to establish possible analogies with humans, with whom they socially interact.

Dogs and humans, like other mammals, have a homologous facial anatomy plan^38,39^ even though they belong to phylogenetically distant groups. Additionally, both species share a common mammalian neuroanatomy for the basic emotions such as fear and happiness^40–44^, typically live in a common social and physical environment, are very facially expressive (e.g.^2,3,20,24^), and respond to the same or similar conspecific and heterospecific social cues (e.g.^45^). Consequently, the facial cues and expression of emotion in home-dwelling pet dogs provide a unique comparative model for the study of phylogenetic inertia (i.e. absence of expected change and/or adaptation to an optimal state given specific selection pressures in the current environment)^46–48^ *versus* evolutionary divergence (i.e. a set of changes brought about by selection pressures from a common ancestor resulting in homologies)^49,50^ *versus* evolutionary convergence (i.e. a set of changes from selection pressures acting in independent lineages to create similarity in the resulting analogies)^47,50^.

Here, we investigated the mechanistic basis of facial expressions in humans and dogs, by objectively measuring their video recorded facial actions during immediate reactions to emotionally-competent stimuli. The FACS^20^ and the DogFACS^24^ were applied in a range of contexts associated with four categories of emotional responses: a) happiness, b) positive anticipation, c) fear, and d) frustration (Table 1). Instead of selecting the basic emotions that are known to produce universal facial signals in humans^51^, we focused on emotions that are defined by evolutionary and biologically consistent criteria: 1) essential for solving adaptive problems in mammals (e.g. fear of a threat prompts flight increasing survival)^52^, 2) arise from corresponding physiological markers (e.g. elevated opioid levels can increase playfulness^53^), and 3) correlate with specific neuroanatomical regions (e.g. Nucleus accumbens neurons activate before a positive event leading to positive anticipation^44,54^). This approach reduces anthropomorphic and anthropocentric bias in the selection and comparison of emotions, i.e. instead of trying to identify stereotypically human emotions in dogs, we focused on examining shared underlying mammalian homologies^55^. Furthermore, for each category of emotion (e.g. fear), we used a range of contexts to generate the emotional response (thunderstorms, specifically avoided objects, etc.). This increased the likelihood of identifying the general facial responses to the emotional category of stimulus (e.g. general facial actions of fear), instead of behavioural motivations (e.g. facial actions displayed for thunderstorms, but not in other fear contexts). We only analysed spontaneous emotional reactions because posed responses could differ from spontaneous ones in duration, intensity, symmetry and form^56–58^.

**Table 1:** Emotion, brain system^44^, definition of emotion, trigger stimuli and context analysed for both species. Different triggers were selected for humans and dogs to allow functional equivalence between species^93,94^.

Emotion	Brain system	Definition	Humans		Dogs	
			Trigger	Context	Trigger	Context
Fear	Fear/anxiety	Aversion/avoidance to a stimulus perceived as a threat, leading to flight, fight, freeze and/or distress response^40,44^.	Visualisation of dangerous animal, experiencing high/fast moving vehicles.	During presentation of animal or experience in a park ride.	Experience of thunderstorms, visualisation of specific objects.	During thunderstorms or presentation of objects.
Frustration	Rage/Anger	Result of denial of a resource by presence of a physical or social barrier, omission/delay of an expected reward, a barrier to success or to achieve a goal^42,103^.	Possibility of gaining a high monetary reward with its subsequent loss(es).	After loss of game/life from the UK TV show game The Cube till start of another attempt/return next to the presenter after game ends.	Visualisation of a desired resource (toy, food, space) that is or becomes inaccessible.	After first attempt to gain access to the resource and during its subsequent denials.
Positive anticipation	Seeking/expectancy	Expectation of potential gain. Desire of a known and predictable resource/goal. Period from signalling of a reward till moment before receiving reward^99,104^.	Visualisation of food, unwrapping a gift.	From visual presentation of food item till moment before eating; From visual presentation of gift till revelation of item.	Visualisation of food or hearing meal/food related word(s); Visualisation of leash, hearing walk related word(s).	After trigger presentation till moment before obtaining food or leaving home.
Happiness	Play/Joy	After/during a positive activity/situation that has a positive outcome for the individual and is intrinsically hedonistic/pleasurable. Only observed in the absence of immediate fitness threats and is highly dependent on all proximate needs being fulfilled^105,106^.	Gain of a high monetary reward.	After win from the UK TV show game The Cube till moment that contestant returns next to the presenter.	Initiation of a play bout; visualisation of owner.	During play with conspecifics or owner; reunion with owner after long period of separation.
Relaxation	—	Absence of any emotionally-linked stimuli and response.	—	—	—	—

Given the common origin of mammalian facial musculature^59^ and its link to emotion^2^, and the nature of the long association between humans and dogs, it is plausible that similar emotional reactions might share common facial correlates in these two species (i.e. that the same emotions are closely associated with the same core facial muscle movements). Therefore, we tested two hypotheses: 1) Do dogs produce specific facial actions in response to different categories of emotionally-competent stimuli (i.e. stimuli that produce an emotion cascade resulting in a distinct brain response^60,61^)? If so, this would provide evidence that dogs possess an adaptive facial behavioural repertoire (*sensu* Schmidt and Cohn^62^) with expressive and/or communicative functions associated with the underlying emotional response, as a result of evolutionary pressures. This is a precondition for the main hypothesis: 2) Do dogs use similar facial muscles to humans to express similar emotions? A lack of significant differences between humans and dogs would potentially reflect phylogenetic inertia and be consistent with the shared basic origin of emotional expression as proposed by Darwin^1^ or reflect convergent evolution. On the other hand, widespread significant differences would indicate that facial expressions of emotion are not highly conserved features across different species.

## Results

### Human facial actions of emotion

Our study showed convergent validity with previous studies (Table 2) for the core AUs^63^ associated with each emotion (Table 1). Humans showed a differential use of their facial musculature to produce a higher rate (i.e. in comparison to the relaxed condition representing neutral facial expression) of specific prototypical facial actions during an emotional response, while using various AUs flexibly between contexts. The comparable core AUs identified in our study included AU5 and AU20 for fear, AU14, AU17 and AU24 for frustration, and AU6 for happiness, confirming our human videos as a suitable baseline to compare with the dog videos. This also allowed us to verify that the naturalistic videos used to extract our data can still produce robust results, with facial signals strongly linked to corresponding emotional responses. Additionally, the human FACS coding created a baseline to compare with the dogs’ responses, based on human-dog muscular homologies.

**Table 2:** Unique human facial movements displayed during different emotional contexts. Significantly different to relaxed context in one context only (p < 0.05, Kruskal-Wallis with post-hoc pairwise comparisons, Dunn-Bonferroni corrected). Unique facial actions found in previous studies are also reported here for comparison purposes, with the respective literature source. H: Test statistic, SE: Standard Error.

Emotion	Humans			Previous studies	Source
	Rate	H	SE		
Fear	AU5 AU7 AU20 AD38	19.700 19.900 24.700 15.500	5.285 5.028 5.285 4.161	AU5, AU20	(54)
Frustration	AU14 AU17 AU24 AU28 AD84	21.100 20.100 21.900 19.600 16.950	5.985 5.920 5.849 5.162 4.555	AU14, AU17, AU24	(20,55,56)
Positive Anticipation	NS	—	—	—	—
Happiness	AU6	33.100	6.308	AU6	(54)

Specifically, our analysis showed that compared to the relaxed condition, humans displayed a significantly higher rate of specific facial actions, in response to stimuli associated with happiness, fear and frustration, but not positive anticipation. For the fear context, the rate of AU5, AU7, AU20 and AD38 were significant; for the frustration context, the rate of AU14, AU17, AU24, AU28 and AD84 were significant; for the happiness context, the rate of AU6 was significant. In the Supplementary Table S4, the facial actions that humans displayed during two or more emotions, but not during the relaxed context are also reported.

### Dog facial actions of emotion

**Figure 1:**
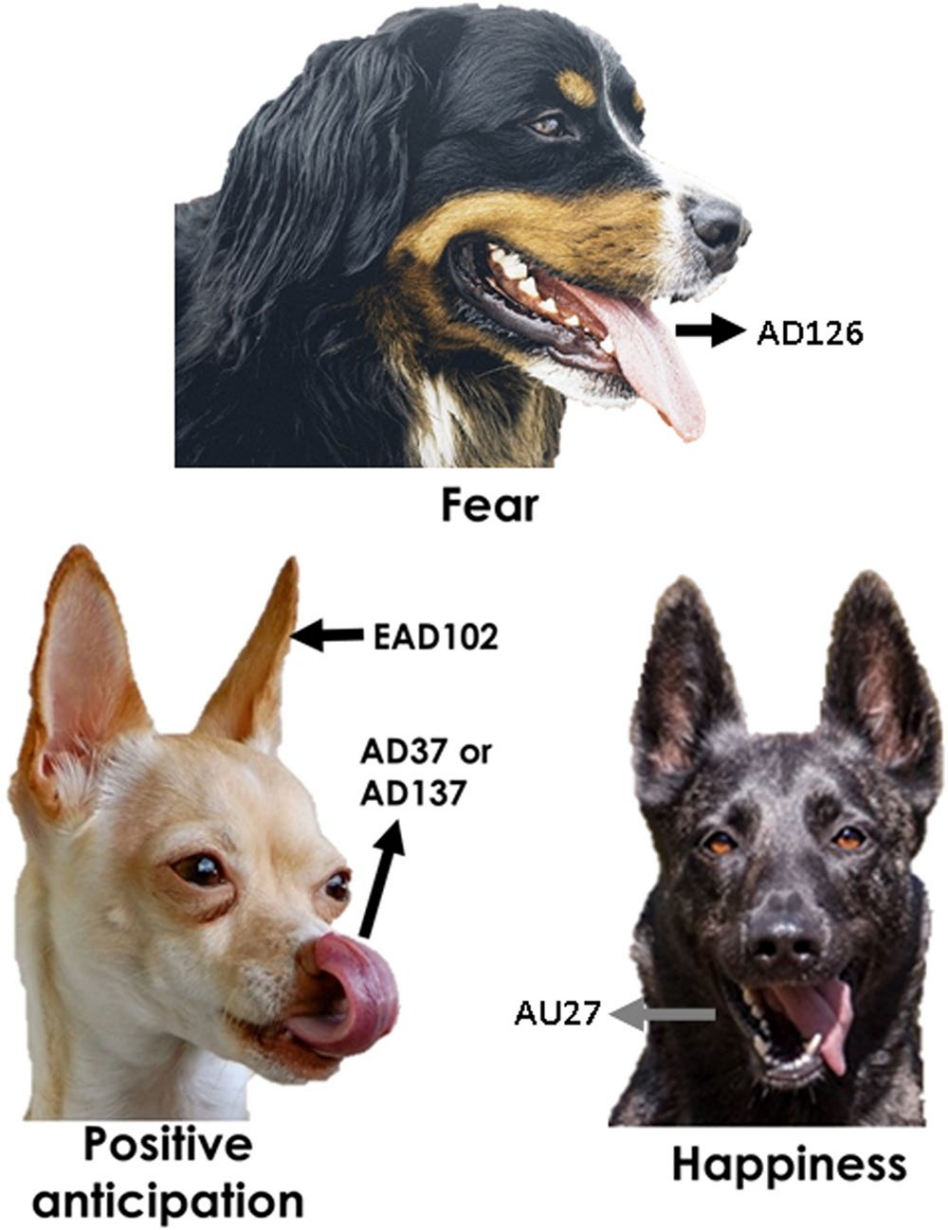
Examples of visual representations of unique dog facial actions displayed during emotional reactions. (Individual images composed for illustration of muscular movements only, found in Pixabay 2016, free for commercial use, https://pixabay.com/en/herder-action-dog-animal-dog-plays-1726406/, https://pixabay.com/en/dogs-cute-pet-animal-canine-1181868/, https://pixabay.com/en/animal-canine-cute-dog-friendly-1837003/).

In support of our first hypothesis, we found significant differences between each emotional response and relaxation for particular facial and ear actions: Dogs consistently showed a higher rate of AD126 during fear contexts; AD37, AD137 and EAD102 during positive anticipation; and AU27 during happiness. However, frustrated dogs did not display higher rates of any of the facial actions (Fig. 1, Table 3). The higher rates of these specific facial actions are thus strongly linked to the respective emotional contexts. In the Supplementary Table S4, we report the display of significantly higher rates of those facial actions common between emotions (but absent during relaxation); like humans, dogs made a clear, flexible use of their facial musculature.

**Table 3:** Unique dog facial movements displayed during different emotional contexts. Significantly different than relaxed context in one context only (p < 0.05, Kruskal-Wallis with post-hoc pairwise comparisons, Dunn-Bonferroni corrected). H: Test statistic, SE: Standard Error.

Emotion	Dogs		
	Rate	H	SE
Fear	AD126	29.300	7.945
Frustration	NS	—	—
Positive Anticipation	AD37 AD137 EAD102	25.200 30.825 24.300	7.263 8.716 8.281
Happiness	AU27	27.600	7.436

### Comparison of human and dog facial actions of emotion

To investigate our second and main hypothesis, direct comparison between human and dog facial actions for each emotion revealed significant differences in specific actions for all the examined emotions (Table 4), demonstrating the differential use of facial musculature between humans and dogs when facing equivalent emotional situations. When compared with humans, dogs displayed higher rates of AD19 in a fearful situation, AU45 during frustration, AD19 and AD37 during positive anticipation, AD19 during happiness, and AU1 in relaxed situations. We also report in Table 4 the facial movements that were significantly higher for humans compared with dogs in the same context. These results show that, in equivalent contexts, dogs and humans mostly activate different facial actions. Out of all the facial actions entered in the analysis, only two Action Units (AU25, AU26) and two Action Descriptors (AD33 and AD40) showed no significant differences between the two species in any of the contexts. This indicates that the majority of facial actions which both dogs and humans are able to produce were used differently for all emotional contexts. Effect sizes were mostly intermediate to large and are reported in the Supplementary Table S5.

**Table 4:** Facial movements that differ between species, within each emotional context (p < 0.05). Bold: higher for dogs, U: Test statistic, SE: Standard Error.

Emotion	Rate	U	SE
Fear	**AD19** AD50 AU10 AU16 AU27 AU43	40.000 178.000 172.000 163.000 170.000 150.000	22.425 20.564 22.517 22.680 20.132 14.759
Frustration	AU43 **AU45** AU10	160.000 167.000 156.500	15.884 22.728 22.517
Positive Anticipation	**AD19 AD37** AU10 AU18	30.000 45.000 168.000 147.000	20.941 19.638 22.588 19.638
Happiness	**AD19** AU10 AU12	36.000 184.000 147.000	21.995 22.588 22.728
Relaxation	**AU1**	35.000	20.564

### Control variables

We found no significant differences between different categories of cephalic type, ear morphology and breed for any of the significant facial actions in our main analysis of dogs, i.e. these variables had no significant impact on the production of facial actions in the emotional contexts analysed in this study. For jowl length, the rate of AU10 during happiness was indistinguishable in dogs with short and long jowls, but was higher than in dogs with no jowls (Kruskal-Wallis, with Dunn-Bonferroni correction, *H*_2_ = 6.736, *p* = 0.050); furthermore AU12 had a significantly higher rate in dogs with short jowls than in dogs with long jowls (Kruskal-Wallis, with Dunn-Bonferroni correction, *H*_2_ = 9.889, *p* = 0.036). However, this observation might be a coding artefact as long or no jowls make these AU movements less conspicuous and thus harder to recognise. Finally, we found significantly higher levels of arousal for all emotions when compared with the relaxed referent (Kruskal-Wallis, with Dunn-Bonferroni correction, Fear: *H*_4_ = 45.20, *p* = 0.0001, frustration: *H*_4_ = 69.650, *p* = 0.0001, positive anticipation: *H*_4_ = 69.857, *p* = 0.0001, happiness: *H*_4_ = 96.500, *p* = 0.0001) and for happiness when compared with fear (Kruskal-Wallis, with Dunn-Bonferroni correction, *H*_4_ = 51.300, *p* = 0.0001). This supports the validity of the relaxed context as a point of reference, since this represents, by definition, absence of/very low arousal.

## Discussion

This study provides the first empirical evidence to address two important research questions in comparative emotion and interspecific emotion understanding: dogs display specific discriminatory facial movements in response to different emotional stimuli, but do not display similar facial movements to humans when reacting to emotionally comparable contexts.

Dogs tended to make more frequent use of AD126 in a fearful context; AD37, AD137 and EAD102 during positive anticipation; and AU27 in a happiness context (Fig. 1). Such observation is in agreement with a widely held belief that dogs facially react in a common way as a species when confronted with particular challenges or situations in their environment (e.g. when fearful^64,65^). This also supports Darwin’s suggestions that facial expressions are correlates of internal states in animals^1^, and are based on homologous facial musculature with the potential to produce similar facial information for a given species. Given previous studies mentioning varied facial cues in frustrated dogs (also described as displacement or conflict behaviours^35,66^), it was surprising that we did not observe distinctive facial actions for frustration in dogs. However, it might be that the frustration signals previously reported are not specific to frustration and are instead used flexibly in association with other behaviours and/or in response to different contexts (e.g. stress^67^, discomfort^68^, fear^65^), and/or that facial expressions of frustration are more context specific, without a common dynamic anatomical structure. Analysing more videos or featuring a wider range of contexts, to account for potential flexibility, might identify specific facial actions for this emotion in certain common contexts.

Regarding our second hypothesis, our results revealed that humans and dogs produced distinct facial expressions of emotion and little commonality was found in the facial actions triggered, i.e., dogs did not show human-like facial expressions. This observation refutes the idea of continuity of emotional expression between different species. Given the clear facial muscular homology and comparable anatomical facial plan between humans and dogs^38,39^, their shared social environment and long history of intensive mutual social interaction^69,70^, it might have been expected that dogs and humans would at least share some facial responses within a comparable emotion context.

Most of the basic facial muscles are similar between humans and dogs, and share primary functions unrelated to facial expression (e.g. the orbicularis oculi muscle that closes the eye to protect the eye or the zygomaticus major that draws the lips back to facilitate feeding). However, when it comes to emotional expression, dogs do not seem to make use of their muscles in the same way that humans do. This might be due to the inability of the dog’s muscles to produce particular movements present in humans because of their different facial morphology (e.g. lack of localised fat deposits). This is the case, for example, with AU6 (produced by the orbicularis oculi muscle), which is a fundamental AU present in all Duchenne happy faces in humans, but in dogs it was never observed even though the same muscle is present and functional. The human face has a more complex facial musculature, i.e. a few muscles are not present in dogs, such as the levator palpebrae that produces AU5 or the risorius that produces AU20. Both AU5 and AU20 (i.e. eyes widely open, lips stretched laterally and downwards) are critical facial actions of a fearful face in humans, but it is impossible for dogs to produce the equivalent facial change.

Another example of muscular variance that might reflect the reported difference in the displayed facial actions relates to the human-dog frustration comparison. Dogs lack a zygomaticus minor muscle, which produces AU14 in humans. This specific movement is one of the core action units of human frustration facial expression. Given the lack of these specific muscles in dogs, it is possible that other muscles could be activated to produce reliable fear or frustration cues in this species. This appears to be the case for fear, since AD126 production had unique characteristics in this context. However, we did not identify an equivalent in the case of frustration, as discussed earlier.

Given the low number of specific facial actions produced in association with each emotion, we suggest that dogs do not display a composed facial expression with several facial actions being integrated in a stereotypical display, as is observed in humans. Instead, dogs seem to produce isolated actions in response to specific emotionally-competent stimuli.

Due to well-known human perception biases (e.g. anthropomorphic tendencies^71^), it is essential to determine in an objective and standardized way exactly how dogs produce emotional cues. The results in relation to our second hypothesis illustrate the error of broad descriptions and over-reliance on holistic categorization of facial behaviour, which can lead to biases in the perception of facial expressions of a different species (e.g.^14,15^). It has been argued that an alternative explanation for why humans perceive non-human animals’ emotions as if conspecifics is because of evolutionary homologies between the species, based on shared mammalian brain structures and behavioural expression patterns^42,71^. However, our study refutes this hypothesis, as the homologies between humans and dogs seem to be limited to the underlying musculature, rather than their activation. In other domestic animals, such as horses^72^ and sheep^73^, the basic facial musculature also appears to be well conserved^26,74^, but the emotional displays appear to diverge from what is observed in humans. It is worth noting that in both of these species (and arguably most domestic animals) the ears appear to be widely used as part of emotional signals, while in humans the ears have more vestigial muscles. Close analysis of the aural signals may therefore be a fruitful line of enquiry in the future. In this respect, it is also worth noting that greater similarities are seen between different species of primates’ (including human) facial expressions^75^, with them showing a continuity not only at a muscular level but also at the level of expression production. In the case of the dog-human comparison, and unlike the chimpanzee-human comparison, facial expressions seem to be exclusive to the species. Phylogenetic inertia was likely involved in the development of the well-conserved facial musculature of dogs and humans^47^, but there are clearly differences in the way this musculature is used to produce facial expressions associated with the intentional or unintentional communication of emotion.

Our findings of humans and dogs displaying distinctively different facial expressions have important theoretical and practical implications on human-dog emotion understanding and its underlying cognitive mechanisms. There is little theoretical understanding of interspecific emotional appraisal, but we examine the extension of two competing theories of human-human emotion perception into the field of human-dog emotion perception: “The mental simulation strategy” (i.e. if the same facial movements are produced in response to the same emotional stimuli in both species, individuals could form an understanding of the emotions of others through their own facial actions), and “The implicit social learning strategy” (i.e. if facial movements produced are different between species, an associative learning between external events and motivational states is likely needed for understanding the emotions of other species)^76,77^. Given that in our study facial movement production differed between humans and dogs, it is unlikely that a mental simulation strategy could be useful or adopted by both species when interpreting heterospecific expressive facial signals. Instead, an implicit social learning strategy would be a more meaningful way to establish a functional human-dog emotion understanding. This would at least partly explain why untrained humans do not seem proficient in reading dogs’ facial and body language^78–80^, particularly subtle cues such as head turning or nose licking^81^. This is further supported by the neurocognitive evidence that people read dogs’ and humans’ social cues using overlapping brain areas^82,83^ and similar cognitive mechanisms^76,84^. Indeed, humans represent non-human animals’ affective space similarly to that of conspecifics’^71,85^ and incorrectly identify emotions in dogs that have been shown to be a direct result of anthropomorphic subjective judgements (e.g. guilt^86^). In our study, most emotional facial actions produced by dogs were in the mouth area, using the tongue or the opening of the mouth, and none were in the eye area, which is an important difference from humans that produce eye region AUs in many of the prototypical facial expressions. Thus this might be another reason why humans seem to find it very hard to read dogs’ communication.

The preceding studies strongly suggest that humans read dog communication as they would read other humans (own-species bias^87^), and our results indicate this is potentially problematic when it comes to evaluation of the emotional content of these signals. This leads to another important issue: all studies to date on human perception of dogs are missing bias-free empirical validation (e.g.^88–90^). For example, when showing a “happy dog” static picture, the only “validation” is the agreement of other subjective human judges, without ever specifying why a “happy dog” is identified as happy, leading to subjective anthropomorphic assessments and circular reasoning, i.e. different human dog “experts” agree with each other on what a “happy dog” should look like, instead of examining what does a “happy dog” actually look like. For the first time, our study demonstrates how to validate dogs’ facial expressions by applying an anatomical and bias-free method based on the presence of empirical stimuli of relevance.

It remains to be seen to what extent the current findings can be generalised across all dog breeds and other emotional contexts, given the variation in facial morphology and possibly muscular functionality in different breeds. Nonetheless, we did establish that features like variation in general head shape may be less important than changes in jowl structure, given the location of key AUs used to express the emotions studied. Despite this study’s focus on facial expressions alone, it is important to mention that emotional expression is clearly multimodal in many species including those studied here (e.g. vocalisations, body posture, and odours may be important moderators of the message sent). While the stimuli were selected for their fundamental emotional equivalence between the two species, the resulting expression might have been moderated by other factors associated with their processing, which were not controlled for (e.g. social vs non-social stimulus source^91^). Nevertheless, the critical feature is that they should largely share a final common emotional process, whose expression we set out to measure. Thus, future studies might aim to compare dogs of different breeds and morphologies, as well as incorporate the multimodality of emotional signals, and test these hypotheses in different settings (e.g. the more controlled laboratory) to expand on these conclusions.

## Methods

### Video source and individuals

The videos used in this study were selected from online databases (www.youtube.com and AM-FED database^92^). Only one individual per video was coded. Whenever there was more than one individual present in the same video that fulfilled all the necessary criteria (see section b), one of the individuals was randomly selected and coded.

The sample consisted of 150 individuals in total, 50 humans and 100 family dogs, distributed equally between emotional contexts (happiness, positive anticipation, fear, frustration and relaxation, Table 1). For detailed information on individuals, please see Supplementary material S1.

### Criteria for video selection

The four categories of emotion were defined according to literature sources (Table 1). As a control, we selected videos of neutral/relaxed individuals, i.e., where any specific emotionally-triggering stimuli or overt behavioural reactions were absent. The videos were chosen on the basis of stimulus quality (e.g. source) and its clear association with an evoked response, with different stimuli selected for humans and dogs to allow functional equivalence^93,94^ (e.g., most humans are not afraid of thunderstorms, while this is a common fear trigger in dogs^95–97^). Videos of dog training, clinical/veterinary settings or with music covering the audio were excluded. Only videos with minimum editing, high image quality (at least 720p), good lighting and visibility of faces were selected. The putative emotion eliciting stimulus had to be apparent and clearly identifiable for at least part of the video. By including homemade/amateur videos with naturalistic and spontaneous behaviour we ensured that the responses were ecologically valid, less constricted, and more robust, especially when compared to laboratory studies on emotions^98^.

To ensure that the emotionally-competent stimuli were present in the dog videos and were impacting their behaviour, the first author of this study (CC) selected, blinded and randomised the video order. Another author (DM, specialist in veterinary behavioural medicine) relabelled all videos according to the emotion likely triggered by the stimulus^99^. Only videos that had full agreement were included in the study.

### FACS, control variables and reliability coding

All human and dog videos were coded with FACS^20^ and DogFACS^24^, respectively, by a certified coder (CC) via the open source software BORIS (Behavioural Observation Research Interactive Software) V2.96^100^. Each video duration was determined by the individual’s emotional response to the trigger, i.e. from the starting of the behavioural display to the ending time point (neutral behaviour). For example, for the fear trigger of a thunderstorm, the trigger was present throughout the video, for all thunderstorm videos analysed, and the duration of the video was considered while the response (flight/freeze, etc.) of the individual was observed. Before starting the FACS coding, one or more neutral face frames were selected for all individuals. The number of facial actions was identified by watching the videos frame-by-frame, logged in BORIS and extracted for analysis. During speech/vocalizations, facial actions were coded only if they were above level A intensity (as per FACS investigator guide recommendation^101^). To ensure an unbiased coding, an independent DogFACS certified coder, unaware of the aim of the study or the emotional categorization of the videos, re-coded a random 30% of the dog videos. Inter-coder reliability was thus obtained with 84% mean agreement in the Intraclass Correlation Coefficient (ICC(3,k), CI range of 0.80–0.87).

Since morphological differences could potentially impact upon the production of facial actions in dogs, we used cephalic type (brachycephalic, mesaticephalic, dolichocephalic), jowls length (none, short, long), ear morphology (floppy, partially erect, erect) and breed (as per the British Kennel Club) as control variables. We only analysed the effects of these control variables in the facial actions that were significant in our main analysis. As an additional control, we categorized all the videos for the perceived level of arousal (five point Likert scale: 1 – very low, 2 – low, 3 – moderate, 4 – high, 5 – very high) to ensure arousal was not a confounding variable for emotion. These control variables allowed us to increase the internal validity of our study, since it excludes alternative explanations for the causal relationship between independent and dependent variables^102^. Different blinded coders recoded the whole sample for each of the control variables to assess mean agreement of inter-rater reliability (ICC(3,k)), obtaining 94% (CI range of 0.91–096) for ears, 85% (CI range of 0.78–0.90), for jowls, and 87% (CI range of 0.81–0.90) for arousal. Human videos were not coded for control or reliability purposes, instead, our results were compared with the respective facial movements reported in the established literature.

### Statistical analysis

Because the videos varied in duration (62 ± 45 s, mean ± s.d.) due to differences in the individuals’ responses to the triggers and the emotions examined, the number of facial actions (4.59 ± 8.97, mean ± s.d.) was normalised into rates of visible time, i.e., the number of each AU was divided by the duration of the video where the face is visible. Variables with very low rates (<0.001) were excluded from analysis (Supplementary material S3). Thus, we included 30 human facial actions and 22 dog facial actions in the statistical analysis (Supplementary Table S2). We performed Kolmogorov–Smirnov tests for normality and non-parametric Levene’s test for homoscedasticity. Since all variables violated both assumptions in at least one of the groups, non-parametric tests were used throughout the analysis. To investigate if dogs produced differential facial actions in response to specific emotional triggers, we compared the facial actions displayed between emotions and between each emotion and the control relaxation videos with Kruskal-Wallis (adjusted for ties) followed by *post-hoc* pairwise multiple comparisons tests, with Dunn-Bonferroni correction. For our second hypothesis, where we were interested in assessing if dogs were using the same facial actions as humans in response to an equivalent emotional trigger, we performed Mann-Whitney tests, with exact p-value for the homologous facial actions between species. For both hypotheses, the rate of facial movements was used as the dependent variable and the emotion category was the independent variable. Additionally, we calculated effect sizes to consider and minimize the risk of type 2 error in our interpretation of the results.

To analyse the potential effect of the control variables cephalic type, ear morphology, jowl length, breed, and arousal level on the facial actions, we used Kruskal-Wallis tests (adjusted for ties) and post-hoc pairwise multiple comparisons tests, with Dunn-Bonferroni correction. Here, we compared the rate of each facial action (dependent variable) between the categories (independent variables) of the control variables, in order to ensure the distribution of facial actions was the same regardless of the dogs’ morphological variation. For ear morphology and jowl length, we only performed the analysis for the relevant facial actions (e.g. jowl length only analysed for lower face actions). All statistical analyses were performed using IBM SPSS v22, except for the effect sizes that were computed with GPower v3.1.

### Ethics

This study was approved by the School of Psychology Research Ethics Committee and follows the Ethics Guidelines for Internet-mediated Research by the British Psychological Society.

### Data availability

The datasets generated and analysed during the current study are available from the corresponding author on request.

## Electronic supplementary material

Supplementary Information

Supplementary Info File 1
