# Author Correction: Dogs and humans respond to emotionally competent stimuli by producing different facial actions

**DOI:** 10.1038/s41598-018-28651-z

**Published:** 2018-07-05

**Authors:** Cátia Caeiro, Kun Guo, Daniel Mills

**Affiliations:** 1 School of Psychology, University of Lincoln, Lincoln, UK; 2 School of Life Sciences, University of Lincoln, Lincoln, UK

Correction to: *Scientific Reports* 10.1038/s41598-017-15091-4, published online 14 November 2017

The original version of this Article contained errors in the spelling of the authors Cátia Caeiro, Kun Guo & Daniel Mills which were incorrectly given as Caeiro Cátia, Guo Kun & Mills Daniel. These errors have now been corrected in the HTML and PDF versions of the Article.

