# Supplementary material for: Dogs and humans respond to emotionally competent stimuli by producing different facial actions
Source: Sci Rep. 2017 Nov 14;7:15525. doi: 10.1038/s41598-017-15091-4 (PMC5686192; doi:10.1038/s41598-017-15091-4)
Supplement: Supplementary file 1 — Supplementary Information [file 41598_2017_15091_MOESM1_ESM.pdf]

Electronic supplementary material:

Article title:

Dogs and humans respond to emotionally competent stimuli by producing different facial actions

Authors:

Caeiro, Cátia[1,2*]; Guo, Kun[1]; Mills, Daniel[2]

[1]School of Psychology, University of Lincoln, UK.

[2]School of Life Sciences, University of Lincoln, UK.

***Corresponding author**:

Cátia C. Caeiro, ccorreaicaeiro@lincoln.ac.uk

S1: The sex and race of the humans coded in the videos were distributed as follows: 25 females and 25 males; 46 Caucasians, 2 Africans, 2 Orientals. The sex and breed of the dogs coded in the videos were distributed as follows: 37 females, 55 males and 8 unknown sex; 34 mixed or unknown breeds, 6 Siberian Huskies, 5 Beagles, 5 Golden Retrievers, 4 Boxers, 3 Alaskan Malamutes, 3 Chihuahuas, 3 Labrador Retrievers, 3 Pitbulls, 2 Cavalier King Charles Spaniels, 2 Cocker Spaniels, 2 French Bulldogs, 2 Dobermans, 2 Greyhounds, 2 Shiba inus, 2 Staffordhsire Terriers, 1 American Eskimo Dog, 1 Australian Cattle Dog, 1 Bernese Mountain Dog, 1 Boston Terrier, 1 Catahoula Leopard Dog, 1 Dutch Shepherd, 1 German Shepherd, 1 Great Dane, 1 Jack Russel Terrier, 1 Pekingese, 1 Pomeranian, 1 Poodle, 1 Pudelpointer, 1 Rottweiler, 1 Samoyedo, 1 Shetland Sheepdog, 1 Vizsla, 1 Weimaraner, 1 Whippet, 1 Yorkshire Terrier. Due to the nature of the videos, age could not be identified, but young children and older adults (estimated age below 16 and above 65 years old) were excluded in order to make the FACS coding more reliable as the age-related facial characteristics of very young children or older adults may impact FACS coding (e.g. wrinkles might be wrongly coded as appearance changes). For the same reasons, we excluded dogs that were either reported by the owners as puppies or that still presented puppy-like facial morphology.

Table S2: Code and description of the facial actions identified in humans[1] and dogs[2] that were entered in the analysis.

Action Units	
**Humans**	**Dogs**
1 Inner brow raiser	101 Inner brow raiser
2 Outer brow raiser	x
4 Brow lowerer	x
5 Upper lid raiser	x
6 Cheek raiser	x
7 Lid tightener	x
43 Eye closure	143 Eye closure
45 Blink	145 Blink
10 Upper lip raiser	110 Upper lip raiser
12 Lip corner puller	12 Lip corner puller
14 Dimpler	x
15 Lip corner depressor	x
16 Lower lip depressor	116 Lower lip depressor
17 Chin raiser	x
18 Lip pucker	118 Lip pucker
20 Lip stretcher	x
23 Lip tightener	x
24 Lip presser	x
25 Lips part	25 Lips part
26 Jaw drop	26 Jaw drop
27 Mouth stretch	27 Mouth stretch
38 Nostril dilator<br>39 Nostril compressor	x

Action Descriptors	
**Humans**	**Dogs**
19 Tongue show	19 Tongue show
28 Lips suck	x
33 Blow	x
37 Lip wipe	37 Lip wipe
x	137 Nose lick
x	126 Panting
40 Sniff	40 Sniff
50 Vocalizations	50 Vocalizations
84 Head shake	x

Ear Action Descriptors	
**Humans**	**Dogs**
x	EAD101 Ears forward
x	EAD102 Ears adductor
x	EAD103 Ears flattener
x	EAD104 Ears rotator
x	EAD105 Ears downwards

Table S3: Common facial movements across contexts, significantly different than relaxed context, but present in more than one context.

Contexts	Humans				Dogs	
	Rate	Proportion	Previous studies	Source	Rate	Proportion
Fear	AU1<br>AU2<br>AU4<br>AU10<br>AU12<br>AU16<br>AU25<br>AU26<br>AU27<br>AD50	AU1<br>AU2<br>AU4<br>AU10<br>AU12<br>AU16<br>AU25<br>AU27<br>AD50	AU1<br>AU2<br>AU4<br>AU25<br>AU26<br>AU27	[3]	AU12<br>AU16<br>AD19<br>AU25<br>AU26<br>AU43	AU12<br>AU16<br>AU25<br>AU26<br>AU43
Frustration	AU1<br>AU2<br>AU4<br>AU10<br>AU25<br>AU26<br>AD50	AU1<br>AU2<br>AU4	AU1<br>AU2<br>AU4	[4–6]	AD19<br>AD50<br>AU16<br>AU25<br>AU26<br>AU43<br>EAD104<br>EAD105	AU43<br>AU25<br>AU26
Positive Anticipation	AU1<br>AU2<br>AU10<br>AU12<br>AD50	AU1<br>AU2<br>AU10<br>AU12<br>AD50	AU12	[7]	AU10<br>AU16<br>AU25<br>AU26<br>AD19<br>AU43<br>AD50<br>EAD103<br>EAD104<br>EAD105	AU16<br>AU25<br>AU26<br>AU43
Happiness	AU10<br>AU12<br>AU16<br>AU25<br>AU26<br>AU27	AU10<br>AU12<br>AU16<br>AU25<br>AU27	AU12	[3]	AU10<br>AU12<br>AU16<br>AU25<br>AU26<br>AD19<br>EAD103<br>EAD104<br>EAD105	AU12<br>AU16<br>AU25<br>AU26

Table S4: Effect sizes (η^2) for Kruskal-Wallis post-hoc tests from first hypothesis and for Mann-Whitney tests from second hypothesis.

Dog Facial Actions	Emotion contrasts	η^2	Effect size
AD19	Relaxation-fear	0.189	large
AU12	Relaxation-fear	0.106	intermediate
AU16	Relaxation-fear	0.125	intermediate
AU25	Relaxation-fear	0.109	intermediate
AU26	Relaxation-fear	0.109	intermediate
AU43	Relaxation-fear	0.121	intermediate
AD126	Relaxation-fear	0.136	intermediate
AD19	Relaxation-positive anticipation	0.123	intermediate
AD37	Relaxation-positive anticipation	0.120	intermediate
AD50	Relaxation-positive anticipation	0.148	large
AU10	Relaxation-positive anticipation	0.085	intermediate
AU16	Relaxation-positive anticipation	0.200	large
AU25	Relaxation-positive anticipation	0.246	large
AU26	Relaxation-positive anticipation	0.237	large
AU43	Relaxation-positive anticipation	0.121	intermediate
EAD102	Relaxation-positive anticipation	0.086	intermediate
EAD103	Relaxation-positive anticipation	0.079	intermediate
EAD104	Relaxation-positive anticipation	0.081	intermediate
EAD105	Relaxation-positive anticipation	0.087	intermediate
AD137	Relaxation-positive anticipation	0.125	intermediate
AD19	Relaxation-happiness	0.121	intermediate
AU10	Relaxation-happiness	0.120	intermediate
AU12	Relaxation-happiness	0.300	large
AU16	Relaxation-happiness	0.184	large
AU25	Relaxation-happiness	0.354	large
AU26	Relaxation-happiness	0.319	large
AU27	Relaxation-happiness	0.137	intermediate
EAD103	Relaxation-happiness	0.093	intermediate
EAD104	Relaxation-happiness	0.099	intermediate
EAD105	Relaxation-happiness	0.100	intermediate
AD19	Relaxation-frustration	0.082	intermediate
AD50	Relaxation-frustration	0.282	large
AU16	Relaxation-frustration	0.086	intermediate
AU25	Relaxation-frustration	0.199	large
AU26	Relaxation-frustration	0.177	large
AU43	Relaxation-frustration	0.121	intermediate
EAD104	Relaxation-frustration	0.182	large
EAD105	Relaxation-frustration	0.215	large

Emotions	Human-dog facial action comparison	η^2	Effect size
Fear	AD19	0.071	intermediate
Fear	AD50	0.143	large
Fear	AU10	0.102	intermediate
Fear	AU16	0.077	intermediate
Fear	AU27	0.120	intermediate
Fear	AU43	0.114	intermediate
Positive anticipation	AD19	0.111	intermediate
Positive anticipation	AD37	0.078	intermediate
Positive anticipation	AU10	0.090	intermediate
Positive anticipation	AU18	0.057	small
Happiness	AD19	0.084	intermediate
Happiness	AU10	0.138	intermediate
Happiness	AU12	0.042	small
Frustration	AU10	0.062	intermediate
Frustration	AU45	0.086	intermediate
Frustration	AU43	0.142	large
Relaxation	AU1	0.099	intermediate

1. Ekman, P., Friesen, W. & Hager, J. *Facial action coding system (FACS): manual*. (Research Nexus, 2002).
2. Waller, B. M. *et al.* Paedomorphic Facial Expressions Give Dogs a Selective Advantage. *PLOS ONE* **8,** e82686 (2013).
3. Ekman, P., Irwin, W. & Rosenberg, E. *The emotional facial action coding system (EMFACS)*. (1994).
4. D'Mello, S. K., Craig, S. D. & Graesser, A. C. Multimethod assessment of affective experience and expression during deep learning. *Int. J. Learn. Technol.* **4,** 165–187 (2009).
5. Grafsgaard, J. F., Wiggins, J. B., Boyer, K. E., Wiebe, E. N. & Lester, J. C. Automatically Recognizing Facial Indicators of Frustration: A Learning-centric Analysis. in 159–165 (IEEE, 2013). doi:10.1109/ACII.2013.33
6. Waller, B. M., Misch, A., Whitehouse, J. & Herrmann, E. Children, but not chimpanzees, have facial correlates of determination. *Biol. Lett.* **10,** 20130974–20130974 (2014).
7. Gubar, G. Recognition of human facial expressions judged live in a laboratory setting. *J. Pers. Soc. Psychol.* **4,** 108–111 (1966).
